# Quantitative analysis of social influence and digital piracy contagion with differential equations on networks

**DOI:** 10.1038/s41598-024-61531-3

**Published:** 2024-05-14

**Authors:** Dibyajyoti Mallick, Kumar Gaurav, Saumik Bhattacharya, Sayantari Ghosh

**Affiliations:** 1 Department of Physics, National Institute of Technology Durgapur, Durgapur, 713209 West Bengal India; 2 Department of Electronics Engineering, Harcourt Butler Technical University, Kanpur, 2082002 Uttar Pradesh India; 3 Department of Electronics and Electrical Communication Engg., Indian Institute of Technology Kharagpur, Kharagpur, 721302 West Bengal India

**Keywords:** Complex networks, Applied mathematics

## Abstract

Illegal file sharing of copyrighted contents through popular file sharing networks poses an enormous threat to providers of digital contents, such as, games, softwares, music and movies. Though empirical studies of network effects on piracy is a well-studied domain, the dynamics of peer effect in the context of evolving social contagion has not been enough explored using dynamical models. In this research, we methodically study the trends of online piracy with a continuous ODE approach and differential equations on graphs to have a clear comparative view. We first formulate a compartmental model to study bifurcations and thresholds mathematically. We later move on with a network-based analysis to illustrate the proliferation of online piracy dynamics with an epidemiological approach over a social network. We figure out a solution for this online piracy problem by developing awareness among individuals and introducing media campaigns, which could be a valuable factor in eradicating and controlling online piracy. Next, using degree-block approximation, network analysis has been performed to investigate the phenomena from a heterogeneous approach and to derive the threshold condition for the persistence of piracy in the population in a steady state. Considering the dual control of positive peer influence and media-driven awareness, we examine the system through realistic parameter selection to better understand the complexity of the dynamics and suggest policy implications.

## Introduction

The term “digital piracy” describes the pervasive issue of unauthorized internet-based copying or dissemination of content protected by copyright. Due to the advancement of recent technologies, any type of digital content may be captured, stored, copied, and altered easily nowadays. Thus, the rise in digital piracy in recent times poses a threat to the growth of creative industries, encompassing publishing, gaming, cinema, television, and music^1,2^. For example, according to the International Federation of Phonographic Industry (IFPI), illicit copying has been a major factor hampering music CD sales, which have dropped by more than 10% yearly since 1997^3,4^. Providing major free-riding apparent benefits to the pirates, digital piracy causes severe detrimental economic issues on government revenue streams. Additionally, it exposes customers to security threats including ID theft and inappropriate content exposure for young audiences^5^

In many studies, it is found that young people are more likely to engage in online piracy as an acquired habit due to a lack of awareness of copyright laws and easy access to the internet^6–9^. However, the perception that piracy is a victimless crime even in the presence of their sense of moral obligation is the most powerful reason behind this^10–13^. Interestingly, the reason for this perception is usually because of their peers who introduce them to piracy^14^. A confirmation from nearest social peers, like parents, siblings, friends etc., about the sustainability of P2P (peer-to-peer) file sharing networks convinces an apparently unaware individuals about high value-to-risk ratio, availability of quality contents and lesser perceived price in digital piracy^15,16^. While these negative peer influence brings them into the arena of piracy, there is also the possibility of positive peer influence that can stop the growth of piracy-related habits. Friends and well-wishers sometimes highlight to youngsters that online piracy is illegal and can have serious consequences^17^. Moreover, to tackle ethical ambiguities, various strategies can be implemented to enlighten individuals about the dangers and repercussions of piracy^18–21^. Initiatives such as education programs, law enforcement efforts, media campaigns, and awareness drives effectively disseminate information regarding the ethical implications of piracy. Along with this, enhancing the affordability and accessibility of content through legal avenues such as OTT platforms can diminish the allure of piracy and serve as a preventive measure^22^. By adopting these approaches, society can work towards curbing the prevalence of piracy while promoting ethical consumption practices^2,23,24^.

Because of its deep economical impacts, understanding the spreading of the habit of piracy is essential to design counter-measures. However, the richness of such a complex dynamics can only be studied with the help of multi-variate dynamical models. It is observed that internet-based spreading mechanisms such as tweeting and sharing online content, like, rumors, gossip, hoaxes, campaigns, etc. have striking similarities with the viral spread of disease, causing epidemic-like spread^25–27^. While one commonly used approach is to study such dynamics using reaction-diffusion methodology^28–31^, epidemic models are also being used recently to study such social contagions. Starting from the very initial SIR models^32,33^, mathematical epidemiology has grown over time, with contributions from various disciplines, like applied mathematics, computer science, social science, and graph theory^34,35^. Beyond conventional empirical studies, considering the epidemic-like spreading of piracy habits from person to person, interesting quantitative approaches^36–39^ have been adopted by researchers to design ways for curbing piracy. In one such study^36^, a model of peer influence for piracy has been explored, where word-of-mouth awareness spreading against piracy has been found to be a possible way to curb it. They have also mentioned a holistic social enlightenment, which, however, cannot be modeled using only the conventional one-to-one awareness model. Instead, it requires a model with more complexity and nonlinearity that includes society-wide awareness campaigns by government agencies^40–42^. In our work we wish to explore this further.

Another important aspect is that the existing studies often consider deterministic ordinary differential equation (ODE) models to analyze such epidemiological dynamics. Such simple ODE models with homogeneous mixing are effective in observing a few critical aspects of complex systems, such as steady states, effects of the various modeling parameters, sensitivity, etc.^43,44^. Although this approach is efficient in providing answers to questions like how many people are affected by a given social contagion at any given time, how many will remain infected in a steady state, the long-term prevalence of infection, and the typology of bifurcation, it does not consider realistic scenarios where a person can interact with a limited number of neighbours. Thus, these models are insufficient for queries about the particular people who are expected to be impacted at any given time or the key players who control the flow of contagion^45^. Moreover, even though the ODE models provide detailed statistics at the population level, they are not very good at explaining information relevant to specific individuals^45–47^. So, to account for the heterogeneity and individual-level information, it is required to study the spreading of epidemic-like piracy on heterogeneous networks^48,49^. To do so, we modify the differential equations for networks, using graph theoretical tools, assuming each individual as a node in the network. Though deterministic and network models both shed light on possible control strategies of pirated content, the relationship between the two approaches needs to be investigated to get deeper understandings. It is important to note that other than some recent exceptions^50,51^, particular case studies on the comparison of homogeneous and heterogeneous approaches of social epidemic models are a less explored area at this point. In this study, we also attempt to relate these two approaches to comprehend the similarities, differences, benefits, and problems of both methodologies^52^. Thus, we organize the manuscript as follows. The respective compartmental model of digital piracy with peer factors and media campaign is proposed in “Model formulation” and analyzed the bifurcations and thresholds mathematically through a deterministic approach^53,54^. Afterwards, we move on with a network-based analysis to illustrate the proliferation of online piracy dynamics over a social network through heterogeneous approach and the numerical simulations on model as well as real networks have been discussed in “Homogeneous analysis”, “Heterogeneous analysis” and “Numerical results”, respectively. The results are summarized by discussing future directions in “Conclusion and discussion”.

**Table 1:** Literature survey supporting transitions of the dynamics.

Respective references	Key observations	Concluded model transitions
Culiberg et al.^55^ , Petrescu et al.^2^ Scaria et al.^56^ Poort et al.^7^ Sameer Hinduja et al.^15^	The major channel for digital piracy is peer-to-peer (P2P) file sharing networks, due its simplicity and ease. Exchanges of media with friends through friendship network is also a feasible possibility. Potential consumers of a certain digital content rely on these networks, sometimes without malicious intent, due to unavailability of movies beyond certain geographical boundaries. Peers using pirated files have a measurable impact on an individual’s inclination towards participating in digital piracy. Based on data from a sample of about 2000 university students, real-life peers who are supportive of unauthorized digital content usage had the largest effect on digital piracy.	$U\longrightarrow B$ transition is driven by Negative Peer Influence
Higgins et al.^17^ Pham et al.^4^ Dörr et al.^57^, Leeuw et al.^23^ Cheung et al.^9^ Gaurav et al.^36^	The role of positive peer influence has been found to be immense as a measure to control piracy. The paid services for accessing movies, software, and music are becoming more popular due to social sharing features and influence of users’ closest peers. An exploration on the factors affecting digital piracy behavior in Vietnam, finds the positive effects of peer influence as a major driver. A quantitative study on evaluating digital policy strategies, programs, and interventions to control the habit takes into account of positive peer influence.	$B\longrightarrow A$ transition is dominated by Positive Peer Influence
Chiang et al.^24^ Niu et al.^39^ Koay et al.^58^	Addiction of restricted content usage on the internet relapses. Willingness to pay for digital music downloads depends on availability of free pirated content. There are possibilities of cue-induced compulsive usage and *cravings* of restricted digital contents Digital piracy intents are impacted by previous history of using pirated goods.	$A\longrightarrow B$ transition is more self-induced in nature, driven by personal cravings.
Park et al.^13^ Leeuw et al.^23^ Sudler et al.^59^ Mirghani^37^	Law enforcement, punishment and campaigns on moral obligation affect online piracy. Current anti-piracy campaigns play a major role in holding back the widespread proliferation of online piracy. Campaigns about strong anti-piracy technology, innovative business models, and piracy analytics can be effective ways to curb piracy.	Importance of media awareness

## Model formulation

### Identifying the transition dynamics

To create a model of the piracy dynamics, we categorized each individual in the society into three compartments: Unaware (U), Bootleggers (B), and Aware (A). The unaware class, denoted by U, is yet to be habituated to online piracy. The bootlegger group of people B consists of individuals who have these online piracy habits and spread these habits through their social contacts. Finally, we have the aware class A, who used to be bootleggers but left the habit of piracy at present, becoming aware of its negative repercussions.

First, to identify the interaction between these subpopulations and the role of their mutual influence on the overall piracy statistics of the entire population, we pin-point specific observations in recent research studies, which are primarily based on thorough surveys. These surveys were conducted to detect the major drivers and barriers in controlling digital piracy, considering particularly the fact that the perceived utility of piracy depends on the consumption habits of peers, directly or indirectly. We highlight their significant findings in Table 1. We detect four essential aspects in terms of behavioral transitions from the cumulative observations:

- Unaware susceptible people may influenced by bootleggers and join them in such activities. Bootleggers convince Unawares about free-rider benefits and this transition occurs with a rate constant $\alpha$.
- Aware people spread awareness against piracy habits among bootleggers and influence them to join class A. Let us take the effective rate as $\rho$.
- A fraction of aware people can not resist themselves to use certain pirated content. This is referred to as *cravings* in the literature. Thus, people from class *A* can again join B class with a relapse rate of $\beta$.
- Government initiatives, such as mass media campaigns of punishments and economic losses, play a significant role in directly curbing piracy.

### Proposed mathematical model

We combine our observations in a dynamical compartmental model with a total population of *T*. Considering the total population $T= 1$ (normalized form),

1 $$\begin{aligned} u+b+a = 1 \end{aligned}$$

For a certain piracy contagion, birth and death can be defined as events that occur when individuals join or depart a specific platform where piracy occurs. To consider a varying demography, where people arrive and exit the population, birth and death rates $\mu$ have been considered here, effectively maintaining a fixed population size. Now, let us consider the external efforts, like global initiatives by governments or international law enforcement organizations, that have been made to bring down the severity of online piracy. Instead of distinguishing different parameters like media campaigns, law enforcement, punishments, advertisements, social drives to boost ethical and moral values, etc., we combine them in a single time-varying parameter. For the sake of simplicity, we will define it as ‘*an effect of media*.’ The Cumulative density of awareness programs driven by the media is denoted by *m*. The differential equation gives the rate of change of media.

2 $$\begin{aligned} \dot{m}=\phi b- \phi _{0}(m-m_{0}) \end{aligned}$$

This equation shows that the extent of media program implementation is assumed to increase linearly with the proportion of bootleggers in the population at the rate $\phi$^41,42,60^. The media awareness program aims to convert the bootleggers into an aware class. Assuming that a particular awareness scheme impacts a specific portion of society, we need more such schemes in the population as the fraction of bootleggers goes higher^35,54^. The depletion rate of these programs due to the ineffectiveness of social barriers in the population is $\phi _{0}$. The society’s awareness level before any awareness program is denoted by $m_{0}$. It shall be noted that *m* is always higher than $m_0$. When $m =m_0$, then $\dot{m} = \phi b$.

The effect of media awareness on bootleggers has been incorporated in the model by introducing a transition from bootlegger to aware at the rate $\gamma m$ where $\gamma$ is the success rate at which a person moves from class *B* to class *A*. In the presence of an external awareness program, the conversion rate from unaware to bootleggers also decreases. This reduction has been incorporated by multiplying $\alpha$ by a factor $(1-\Theta )$ where $\Theta$ is $\frac{m}{c+m}$. The positive constant *c* limits the effect of awareness programs on unawares. For finite *c*, as media approaches $\infty$, $\Theta$ approaches the saturation point of 1. The $\Theta$ attains the half-saturation point of $\frac{1}{2}$ when *m* is equal to *c*. That’s why the constant *c* is known as the half-saturation point for Holling type-II functional response^60^. A schematic diagram representing all the transitions between different classes has been shown in Fig. 1. Considering the transition rates to be strictly positive real numbers, the coupled differential equations for the model are as follows:

3 $$\begin{aligned} \dot{u}= & {} \mu -\alpha (1-\Theta ) u b -\mu u,\nonumber \\ \dot{b}= & {} \alpha (1-\Theta ) u b-\rho b a+\beta a -\gamma m b -\mu b,\nonumber \\ \dot{a}= & {} \rho b a-\beta a +\gamma m b - \mu a, \nonumber \\ \dot{m}= & {} \phi b- \phi _{0}(m-m_{0}) \cdot \end{aligned}$$

## Homogeneous analysis

**Figure 1:**
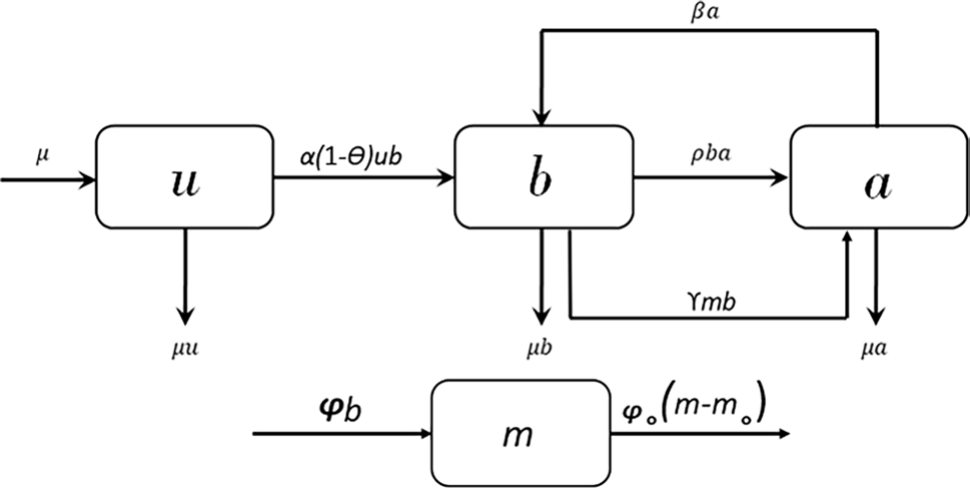
Block diagram of the proposed model for the propagation of habit of online piracy in the presence of mass media awareness.

### Equilibrium analysis

After attaining the steady state, the rate of change of all the subpopulations, *u*, *b*, and *a*, must be zero. Solving the Eq. set 3, to zero, we get

4 $$\begin{aligned} m^\star =m_{0}+zb^\star ; \; u^\star ={\scriptstyle { \frac{(c+m_{0}+zb^\star )\mu }{(c+m_{0}+zb^\star )\mu +\alpha c b^\star }}} ; \; a^\star ={\scriptstyle {\frac{(m_{0}+zb^\star )\gamma b^\star }{\beta +\mu -\rho b^\star }}} \end{aligned}$$

where $z=\frac{\phi }{\phi _{0}} \cdot$ Substituting the values of $u^\star$ and $a^\star$ in $(u^\star +b^\star +a^\star =1)$, we get a cubic equation in $b^\star$ i.e.,

$$p (b^{\star })^{3}+q (b^{\star })^{2}+r b^\star =0$$ where,

5 $$\begin{aligned} p= & {} (\mu z+\alpha c)(\gamma z-\rho ), \nonumber \\ q= & {} (\mu z+\alpha c)(\beta +\mu +m_{0} \gamma )+\mu (c+m_{0})(\gamma z-\rho ) +\alpha c \rho , \nonumber \\ r= & {} \mu (c+m_{0})(\beta +\mu +m_{0} \gamma )-\alpha c (\beta +\mu ) \cdot \end{aligned}$$

From the equation, it can be observed that $b^\star =0$ is always a solution. The respective values of $u^\star$ and $a^\star$ are 1 and 0. Hence, $E_{0}(1, 0, 0)$ is always a steady-state solution of the system. To investigate other roots, we consider $b^\star \ne 0$ and are left with quadratic equation $p (b^{\star })^{2}+q b^{\star }+r = 0$, where on the basis of sign of the coefficient *p* there are two different situations:

**Case 1**: $p>0$ implies $\gamma z>\rho$, which in turn also implies $q>0.$ There will be one (or no) positive solution depending on whether *r* is negative (or positive). Hence, the required condition to have a positive solution of the quadratic equation is

$$\begin{aligned} \alpha c (\beta +\mu )> \mu (c+m_{0})(m_{0} \gamma +\beta +\mu )\cdot \end{aligned}$$

It expresses the reproduction number $\mathscr {R}_{m}.$ A single endemic steady state exists only when

6 $$\begin{aligned} \mathscr {R}_{m}=\frac{\alpha c (\beta +\mu )}{\mu (c+m_{0})(\beta +\mu +m_{0} \gamma ) }>1 \cdot \end{aligned}$$

otherwise, only a piracy free steady state $E_{0}$ exists.

**Case 2**: When $p<0$ i.e, $\gamma z<\rho .$ Depending on the sign of *r*, this case can be divided into two sub-cases.

**Sub-case 2.1**: When $r>0$ ($\mathscr {R}_{m}<1$), similar to Case 1, one of the roots is positive, and the other is negative.

**Sub-case 2.2**: When $r<0$ ($\mathscr {R}_{m}>1$), *q* will surely be positive. It can be observed after rearranging the expression of *q* and *r* in Eq. set 5.

$$\begin{aligned} q= & {} (\mu z+\alpha c)(\beta +\mu +m_{0} \gamma )+\mu (c+m_{0})\gamma z - \rho (\mu c+\mu m_{0}-\alpha c), \nonumber \\ r= & {} \mu (c+m_{0})(m_{0} \gamma )+(\beta +\mu )(\mu c+\mu m_{0}-\alpha c)\cdot \end{aligned}$$

The term is responsible for a negative sign of *r*, i.e., $(\mu c+\mu m_{0}-\alpha c)$ appears in the expression of *q* with a negative sign resulting in positive *q*. With $p<0$, $q>0$, and $r<0$, it is ensured that both roots of the quadratic equation will be positive.

Quadratic equation in $b^{\star }$ tells that there will be one or two positive solutions when ($\mathscr {R}_{m}<1$) or ($\mathscr {R}_{m}>1$), but it does not guarantee that all these solutions will be physical (i.e., $0\le b^\star \le 1$). To investigate it further, along with the quadratic equation in $b^\star$, we have also analyzed a quadratic equation in $a^\star$. All three classes’ change rates will be zero at a steady state. Equating the third equation of the coupled equation, Eq. (3) to zero, we get

$$\begin{aligned} \rho b a-\beta a +\gamma m b - \mu a =0 \cdot \end{aligned}$$

At steady state, value of *b*, *a*, and *m* will be $b^\star$, $a^\star$, and $m^\star$ respectively. Replacing $b^\star$ by $(1-u^\star -a^\star )$ and substituting $m^\star$ and $a^\star$ from Eq. (4), we get a quadratic equation $p_{a}(a^\star )^{2}+q_{a}a^\star +r_{a}=0$ where

7 $$\begin{aligned} p_{a}= & {} (\gamma z-\rho ),\nonumber \\ q_{a}= & {} (\rho -2z\gamma )(1-u^\star )-\gamma m_{0} -(\mu +\beta ),\nonumber \\ r_{a}= & {} \gamma m_{0}(1-u^\star )+\gamma z-\gamma z u^\star (2-u^\star )\cdot \end{aligned}$$

Based on the above discussion, we conclude that there exists only one physical endemic steady state beyond $\mathscr {R}_{m}>1$.

For $\mathscr {R}_m<1$, examining the roots of quadratic equations in $b^\star$ and $a^\star$, it can be observed that one of the roots is positive and another is negative in both cases. For any physical solution, $u^\star$, $b^\star$, and $a^\star$ must be individually in the range [0,1]. For $0\le u^\star <1$, quadratic equation in $b^\star$ and $a^\star$ gives two roots ($b^{\star }_1, b^{\star }_2$) and ($a^{\star }_1, a^{\star }_2$) respectively which finally form steady state triplets $(u^{\star }_1, b^{\star }_1, a^{\star }_1)$ and $(u^{\star }_2, b^{\star }_2, a^{\star }_2)$. For $\mathscr {R}_m<1$, if $b^{\star }_1$ is positive then $b^{\star }_2$ is negative or vice versa. As discussed earlier, the statement also holds for $a^{\star }_1$ and $a^{\star }_2$ in this range. To check the feasibility of the triplets, let us assume that $(u^{\star }_1, b^{\star }_1, a^{\star }_1)$ is physical, i.e., $u^{\star }_1$, $b^{\star }_1$, and $a^{\star }_1$ are in the range 0 to 1 individually, and $u^{\star }_1+b^{\star }_1+a^{\star }_1=1$. However, if our assumption is true, it implies that $b^{\star }_2$ and $a^{\star }_2$ will surely be negative. But, for $0\le u^{\star }_2 < 1$, along with negative values of $b^{\star }_2$ and $a^{\star }_2$, the required condition of $u^{\star }_2+b^{\star }_2+a^{\star }_2=1$ will never hold. Hence, our initial assumption that both $b^{\star }_1$ and $a^{\star }_1$ are positive, is not right. Actually, for positive $b^{\star }_1$, $a^{\star }_1$ will be negative and for negative $b^{\star }_1$, $a^{\star }_1$ will be positive. Hence, we conclude that for $\mathscr {R}_m<1$, neither of the endemic states is physical, and only piracy-free steady-state prevails.

We conclude that the scenario is the same whether $\gamma z <\rho$ or $\gamma z > \rho$. For $\mathscr {R}_{m}<1$, there exists only a piracy-free steady state, and for $\mathscr {R}_{m}>1$, there exists a unique endemic steady state.

**Sensitivity analysis:** The value of $\mathscr {R}_m$ depends on $\alpha$, *c*, $\beta$, $\mu$ $m_0$, and $\gamma$. For any parameter *x* the sensitivity of $\mathscr {R}_m$ is defined as

8 $$\begin{aligned} \zeta ^{\mathscr {R}_m}_{x}=\frac{x}{\mathscr {R}_m}\cdot \frac{\partial \mathscr {R}_m}{\partial x} \end{aligned}$$

Using this definition, sensitivities of $\mathscr {R}_m$ for all six parameters are as follow:

$$\begin{aligned} \zeta ^{\mathscr {R}_m}_{\alpha }= & {} 1,\;\;\; \zeta ^{\mathscr {R}_m}_{c}=\frac{m_0}{c+m_0}, \;\;\; \zeta ^{\mathscr {R}_m}_{\beta }=\frac{\beta }{\beta +\mu }\cdot \frac{m_0 \gamma }{\beta +\mu +m_0 \gamma }, \nonumber \\ \zeta ^{\mathscr {R}_m}_{m_0}= & {} -\Bigg (\frac{m_0 \gamma }{\beta +\mu +m_0 \gamma }+ \frac{m_0}{c+m_0}\Bigg ), \;\;\; \zeta ^{\mathscr {R}_m}_{\mu }=-\Bigg (\frac{\beta }{\beta +\mu }+\frac{\mu }{\beta +\mu +m_o \gamma }\Bigg ), \;\;\; \zeta ^{\mathscr {R}_m}_{\gamma }=-\frac{m_0 \gamma }{\beta +\mu +m_0 \gamma }\cdot \end{aligned}$$

### Bifurcation and physical interpretation

**Figure 2:**
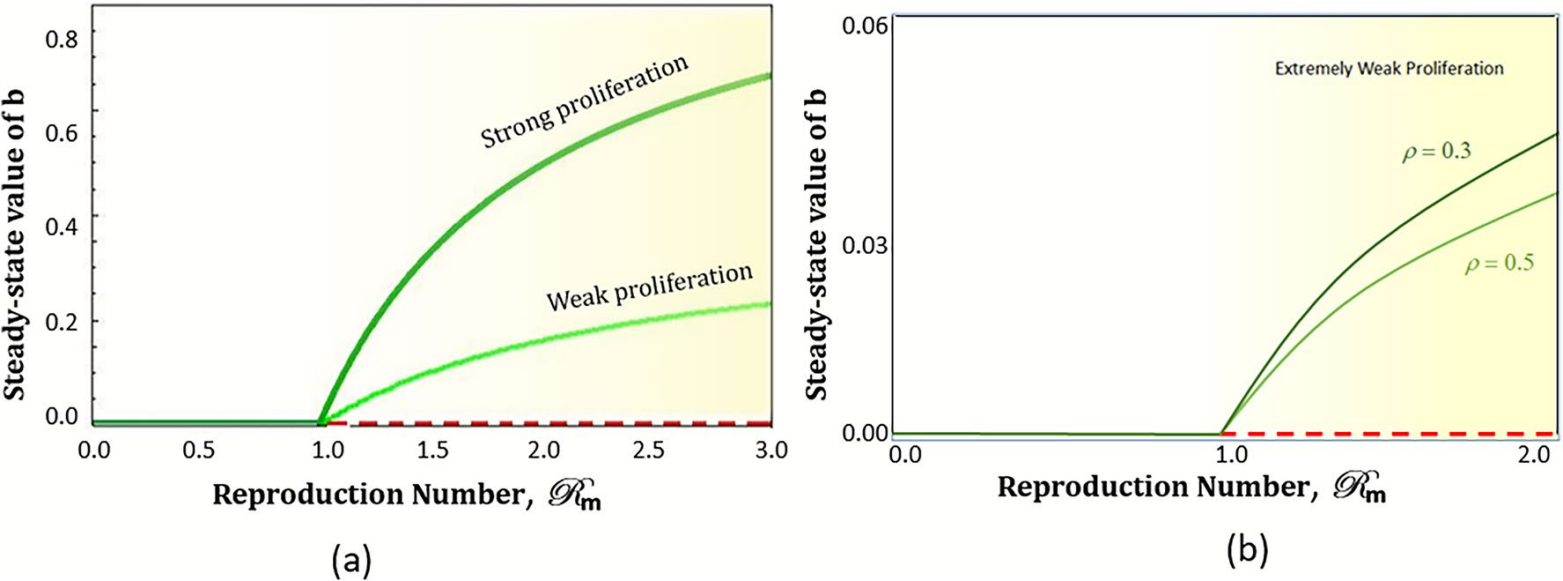
Variation of reproduction number $\mathscr {R}_{m}$ with the steady-state fraction of *b* (**a**) Media effect: the green line represents no media while the light green line shows the presence of media. (**b**) For two values of $\rho$, i.e., 0.3 and 0.5. Other parameter values are $\mu =0.05$, $\beta = 0.2$, $\gamma = 0.08$, $\phi = 0.05$, $\phi _{0}= 0.01$, $c=5$ and $m_{0}=4$. Green lines indicate stable solutions in these figures, and red lines indicate unstable solutions.

The bifurcation diagram for the system is shown in Fig. 2. A forward transcritical bifurcation is observed at $\mathscr {R}_{m}=1$, where a new endemic steady state appears and the endemic free equilibrium loses its stability. For comparison, we also analyze the bifurcation for $m=0$ and $\phi =0$ as shown in Fig. 2a. Regarding physical interpretation, the most crucial difference we observe here is the drastic decrease in the bootlegger population at the steady state. In the presence of mass media campaigns, the value of $b^{\star }$ becomes nearly 15% for the considered parameter values, which are very small compared to the model without media. With media, the fraction of people interested in piracy, even for higher values of reproduction number, remains substantially less, which can successfully reduce the adverse effects of the piracy epidemic. We denote this mass-level control strategy as *weak proliferation*, an excellent and realistic way to handle the piracy epidemic. The bifurcation diagram for two different values of $\rho$ has been shown in Fig. 2b, and it can be observed that the steady-state fraction of bootleggers decreases further with an increase in awareness parameter $\rho$. This establishes that this mass-media-driven law enforcement seems to be a very efficient control strategy for a habit like online piracy, which has a very high degree of induction due to straightaway economic benefits to the users.

### Effect of mass media

As discussed in the previous section, system behavior depends on the value of $\mathscr {R}_{m}$, which in turn depends on other model parameters. To understand the effect of media on the process, we have investigated the individual contribution of parameters *c* and $m_{0}$, which control the effect of the media campaign.

#### Effect of *c*

**Figure 3:**
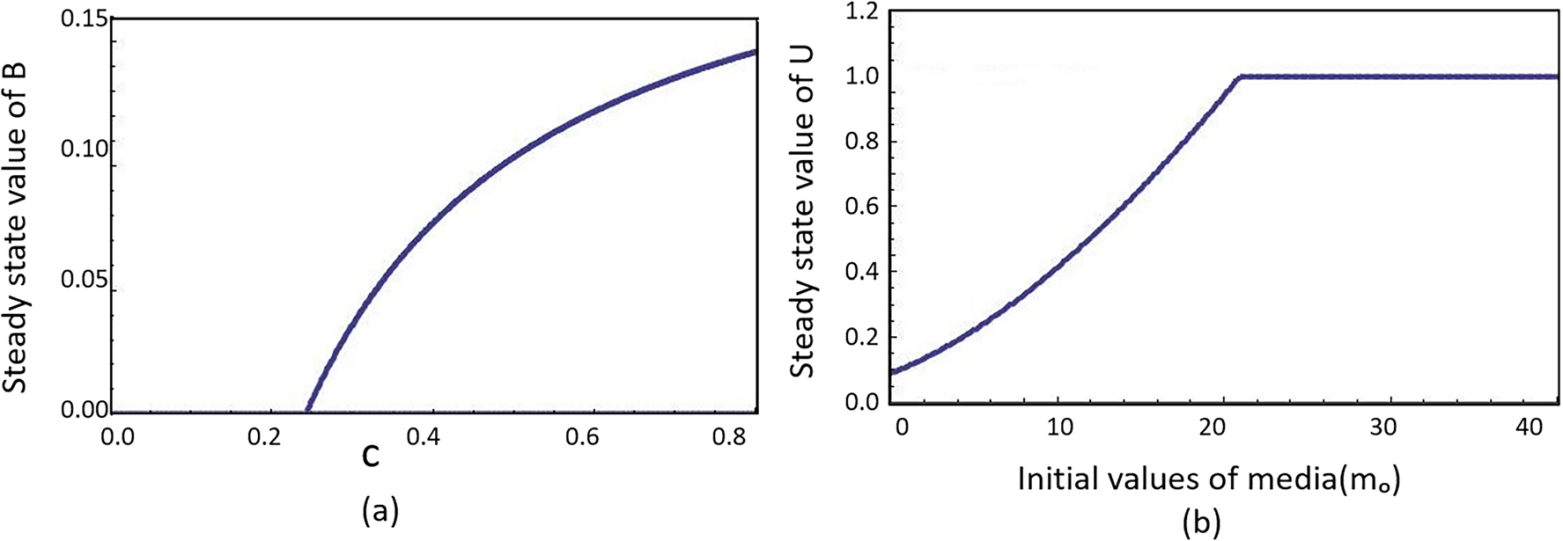
Variation in steady state fraction of (**a**) bootleggers with constant *c*, which in turn changes the value of $\Theta$ and decides the rate of conversion from class *U* to *B* and (**b**) unaware class with the different initial level of intrinsic social awareness $m_0$. Parameters having same value in both the cases are $\mu =0.05$, $\alpha =2$, $\beta =0.2$, $\rho =0.3$, $\gamma =0.08$, $\phi =0.5$, and $\phi _{0}=0.1$. In (**a**) $m_0=4$ and in (**b**) $c=5$. Threshold $c_{th}$ in (**a**) is 0.24178 and $m_{0_{th}}$ in (**b**) is 20.955.

The parameter *c* controls the value of $\Theta$, which modifies the conversion rate from class *U* to class *B* in the presence of mass media awareness. For piracy habit to become extinct from the population, the required condition is $\mathscr {R}_{m}<1$, i.e., $\mathscr {R}_{m}=\frac{\alpha c (\beta +\mu )}{\mu (c+m_{0})(\beta +\mu +m_{0} \gamma ) }<1$. Rearranging the above equation, we get the threshold condition on the value of *c* as $c<\frac{m_{o}\mu }{(\alpha -\mu )-(\frac{\alpha m_{0}\gamma }{\beta +\mu +m_{0} \gamma })}$. The right-hand side of the inequality is denoted by $c_{th}$. As shown in Fig. 3a, for $c<c_{th}$ value of *b* is 0, which means that the complete population is in an endemic free state and the problem of piracy does not exist. For *c* greater than $c_{th}$, a non-zero value of *b* can be observed, signifying the presence of people indulging in piracy.

#### Effect of $m_{0}$

Analyzing the effect of intrinsic level of social awareness $m_{0}$, we get the following quadratic expression from the threshold condition $\mathscr {R}_{m}<1$

9 $$\begin{aligned} \mu \gamma m_{0}^2+\mu (\beta +\mu +c\gamma )m_{0}+c(\beta +\mu )(\mu -\alpha )>0 \cdot \end{aligned}$$

For $(\mu -\alpha )>0$, both roots of $m_{0}$ will be negative. For any positive value of $m_{0}$, the required condition for the extinction of piracy will hold. For $(\mu -\alpha )<0$, one of the roots will be positive, and that will be the threshold value of $m_{0}$, denoted by $m_{0_{th}}$. If the value of intrinsic social awareness is more than this positive root of the quadratic equation, society will be free from piracy. In Fig. 3b, we can observe that beyond this threshold value, a fraction of unaware becomes 1, indicating the absence of piracy.

## Heterogeneous analysis

Though the homogeneous model predicts the system’s steady state, it does not give any information about the time evolution of a particular node. In this section, we will analyze the transmission of piracy habits in heterogeneous populations, incorporating its network structure in the differential equation model.

### Degree block approximation

In terms of density functions, Eq. (3) can be written as

10 $$\begin{aligned} u_k'= & {} \mu -\alpha _{n} k (1-\Theta ) u_{k} \psi _{b}-\mu u_{k}, \nonumber \\ b_k'= & {} \alpha _{n} k (1-\Theta ) u_{k} \psi _{b}-\rho _{n} k b_{k} \psi _{a}+\beta a_{k}-\gamma m b_{k}-\mu b_{k}, \nonumber \\ a_k'= & {} \rho _{n} k b_{k} \psi _{a}-\beta a_{k} +\gamma m b_{k} -\mu a_{k}, \nonumber \\ m'= & {} \phi b- \phi _{0}(m-m_{0})\cdot \end{aligned}$$

Piracy contagion in networks will depend on the network’s degree distribution, as opposed to the homogeneous method. Hence, In a heterogeneous setting, *u*, *b*, and *a* have been modified to $u_k$, $b_k$, and $a_k$ as the fraction of unaware, bootlegger, aware nodes having degree *k*. And besides, here we used the rate parameters as $\rho _n$ and $\alpha _n$ instead of $\rho$ and $\alpha$ of heterogeneous analysis. The value of $\rho _n$ should be the average piracy habit spread in the heterogeneous approach, equal to the overall spread by a bootlegger in the homogeneous approach. However, the impact of mass media has been considered the same for all nodes, irrespective of their degrees. That’s why in the expression of $m'$, we have used *b*, not $b_k$. Here, *b* signifies the fraction of bootleggers in the entire population. Multiplying the first three equations of the coupled equation, Eq. (10) by $\frac{kp_{k}}{\langle k \rangle }$ and summing over *k*, we get

11 $$\begin{aligned} \psi _{u}'= & {} \mu -\alpha _{n} \sum _{k} \frac{k^{2} p_{k}}{\langle k \rangle }u_{k} \frac{c}{c+m}\psi _{b}-\mu \psi _{u}, \nonumber \\ \psi _{b}'= & {} \alpha _{n} \sum _{k} \frac{k^{2} p_{k}}{\langle k \rangle }u_{k} \frac{c}{c+m} \psi _{b}-\rho _{n} \sum _{k} \frac{k^{2} p_{k}}{\langle k \rangle }b_{k} \psi _{a}+\beta \psi _{a}-\gamma m \psi _{b} -\mu \psi _{b}, \nonumber \\ \psi _{a}'= & {} \rho _{n} \sum _{k} \frac{k^{2} p_{k}}{\langle k \rangle }b_{k} \psi _{a}-\beta \psi _{a}+ \gamma m \psi _{b} -\mu \psi _{a} \cdot \end{aligned}$$

Here $p_k$ is the fraction of nodes in the network with degree *k*, and $\langle k \rangle$ is the average degree.

### Early stage analysis

Analyzing the dynamics from the early stage, where very few people are aware of using pirated versions, we are approximating $u_{k}$ by 1, and $b_{k}$ as well as $a_{k}$ is considered to be negligible. This approximation is used to linearize the nonlinear terms, Eq. (11) is simplified to

12 $$\begin{aligned} \psi _{u}'= & {} \mu -\alpha _{n} \frac{\langle k^{2} \rangle }{\langle k \rangle } \frac{c}{c+m}\psi _{b}-\mu \psi _{u}, \nonumber \\ \psi _{b}'= & {} \Bigg (\alpha _{n} \frac{\langle k^{2} \rangle }{\langle k \rangle }\frac{c}{c+m}-(\gamma m +\mu )\Bigg )\psi _{b}+\beta \psi _{a}, \nonumber \\ \psi _{a}'= & {} -(\beta +\mu ) \psi _{a}+ \gamma m \psi _{b} \cdot \end{aligned}$$

The last two equations of the coupled equations, Eq. (12), form a system of simultaneous linear differential equations with constant coefficients.

13 $$\begin{aligned} \psi _{b}'= & {} C_{1}\psi _{b}+C_{2} \psi _{a},\;\;\;\; \psi _{a}' = C_{3} \psi _{a}+ C_{4} \psi _{b} \cdot \end{aligned}$$

We observe that the necessary condition for initial growth in class *B* is $C_{1}C_{3}<C_{2}C_{4}$^61^. Substituting the expression of all these constant terms, the condition modifies to

$$\begin{aligned} \frac{\alpha _{n}}{\mu } \Bigg (\frac{c}{c+m}\Bigg )\Bigg (\frac{\beta +\mu }{\beta +\mu +\gamma m}\Bigg )>\frac{\langle k \rangle }{\langle k^{2} \rangle } \cdot \end{aligned}$$

Replacing $\alpha _n$ by $\frac{\alpha }{\langle k \rangle }$, we get

$$\begin{aligned} \frac{\alpha }{\mu } \Bigg (\frac{c}{c+m}\Bigg )\Bigg (\frac{\beta +\mu }{\beta +\mu +\gamma m}\Bigg )= \mathscr {R}_m>\frac{\langle k \rangle ^2}{\langle k^{2} \rangle } \cdot \end{aligned}$$

The right-hand side of the inequality is *epidemic threshold* in terms of network parameters. The first and second moments of degree distribution will depend on the network structure.

**Figure 4:**
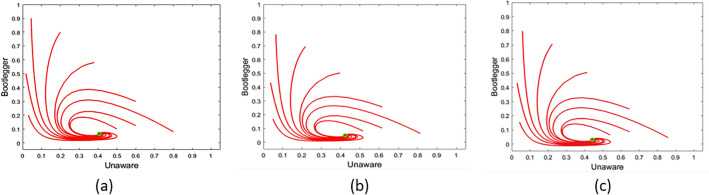
Temporal variation of *u* and *b* in case of an endemic steady state with different initial conditions for the parameter set $\mu = 0.05$, $\alpha = 2$, $\beta = 0.01$, $\rho = 0.3$, $\gamma = 0.08$, $m_0=4$, $c=5$, $\phi =0.5$ and $\phi _0=0.1$ in case of (**a**) homogeneous setting; (**b**) random network; (**c**) Jazz network. The value of $\mathscr {R}_m$ for the considered parameter set is 3.5, which is greater than 1.

### Steady state analysis

In steady state, rate of change of $u_{k}$, $b_{k}$, $a_{k}$ and *m* will be zero. Equating the last equation of the coupled Eq. (10) to zero, we get $m=m_{0}+zb$ where $z=\frac{\phi }{\phi _{0}}$. Using this expression of *m* in Eq. (10), we get

$$\begin{aligned} u_{k}={{\frac{\mu (c+m_{0}+bz)}{\mu (c+m_{0}+bz)+\alpha _{n} k c \psi _{b}}}} ; \ a_{k}={{\frac{\rho _{n} k \psi _{a}+ \gamma (m_{0}+bz)}{\beta +\mu }}}b_{k}\cdot \end{aligned}$$

Substituting these expressions of $u_{k}$ and $a_{k}$ in $( u_{k}+b_{k}+a_{k}=1)$, we get

$$\begin{aligned} b_{k} ={{\Bigg (\frac{\beta +\mu }{\beta +\mu +\rho _{n} k \psi _{a}+\gamma (m_{0}+bz)}\Bigg )\Bigg (\frac{\alpha _{n} k c \psi _{b}}{\alpha _{n} k c \psi _{b}+\mu (c+m_{0}+bz)}\Bigg )}}\cdot \end{aligned}$$

Multiplying above equation by $\frac{kp_{k}}{\langle k \rangle }$ and summing over *k* we get

14 $$\begin{aligned} \psi _{b} = \sum _{k} \frac{ k p_{k}(\beta +\mu )\alpha _{n} k c \psi _{b} }{{\{\langle k \rangle (\beta +\mu +\rho _{n} k \psi _{a}+ \gamma (m_{0}+bz)) \quad ( \alpha _{n} k c\psi _{b}+\mu (c+m_{0}+bz))\}}}\cdot \end{aligned}$$

This is a self-consistency equation of the form $\psi _{b}=f(\psi _{b}, \psi _{a})$ having 0 as an obvious solution. At $\psi _{b}=1$, which also implies $b=1$

15 $$\begin{aligned} f(1, \psi _{a}) = \sum _{k} \frac{k p_{k}(\beta +\mu )(\alpha _{n} k c)}{{\{\langle k \rangle (\beta +\mu +\rho _{n} k \psi _{a}+\gamma (m_{0}+z)) ( \alpha _{n} k c + \mu (c+m_{0}+z))\}}}\cdot \end{aligned}$$

Observing the numerator and denominator of the Eq. (15), we can see that for every term in the numerator, there is a corresponding larger term in the denominator. Hence, $f(1, \psi _{a})$ will surely be less than 1. To have a solution of Eq. (14) in the interval $\psi _{b}=(0,1)$, slope of the function *f* must be greater than 1 at the point $(\psi _{b}=0, \psi _{a}=0)$. The slope of the function is

$$\begin{aligned} \frac{\partial f(\psi _{a},\psi _{b})}{\partial \psi _{b}}= & {} \frac{(\beta +\mu )\alpha _{n} c}{\langle k \rangle (\beta +\mu +\rho _{n} k \psi _{a}+\gamma (m_{0}+bz))}\times \sum _{k} \frac{\mu (c+m_{0}+bz) k^{2} p_{k}}{(\mu (c+m_{0}+bz)+\alpha _{n} k c\psi _{b})^{2}}\cdot \end{aligned}$$

At point $(\psi _{b}=0, \psi _{a}=0)$, value of the slope is

$$\begin{aligned} \frac{(\beta +\mu )\alpha _{n} c \langle k^{2} \rangle }{(\beta +\mu +\gamma m_{0})\mu (c+m_{0}) \langle k \rangle }\cdot \end{aligned}$$

Hence, the required condition to have a desired solution for the Eq. (14) is

$$\begin{aligned} \frac{\alpha _{n} (\beta +\mu )c }{\mu (\beta +\mu +\gamma m_{0})(c+m_{0}) }>\frac{\langle k \rangle }{\langle k^{2} \rangle } \cdot \end{aligned}$$

Substituting $\alpha _n$ by $\frac{\alpha }{\langle k \rangle }$, the condition is equivalent to $R_{m}>\frac{\langle k \rangle ^2}{\langle k^{2} \rangle }$ Which is the same as obtained in “Early stage analysis”.

## Numerical results

In this section, we will discuss the results of the spread of piracy habits after applying mass media awareness in the synthetic society. We will compare the results of the homogeneous and heterogeneous analysis. In the heterogeneous part, results over real networks will also be discussed along with model networks.

### Simulation of homogeneous model

In “Bifurcation and physical interpretation”, we found that after mass media awareness, people with a habit of piracy will exist only beyond $\mathscr {R}_m=1$. If we consider the reproduction number without media campaigns, $\mathscr {R}_m$, includes two multiplicative factors: $\frac{\beta +\mu }{\beta +\mu +\gamma m_0}$ and $\frac{c}{c+m_{0}}$, which are always less than 1. Hence, effective implementation of mass media awareness programs makes the epidemic threshold comparatively difficult to cross, and the endemic regime less accessible. Temporal evolution in the $u-b$ plane for different initial points have been shown in Fig. 4a, for $\mathscr {R}_m>1$. We can observe that, at the steady state, the fraction of bootleggers in the population is meager.

**Table 2:** Steady state values of different classes for different networks in the presence of mass media awareness.

Steady state fraction	Homogeneous setting	Random network	Jazz network
$u^{\star }$	0.407	0.428	0.452
$b^{\star }$	0.061	0.060	0.058
$a^{\star }$	0.507	0.483	0.402

### Simulation over model networks

Similar to homogeneous cases, we can notice a significant decrease in the steady state fraction of bootleggers in the case of model networks as well. Temporal evolution for the random network has been shown in Fig. 4b. The steady-state value in the case of a random network simulation is similar to the homogeneous scenario. Error in the steady-state fractions of different classes is bounded by 2% for the considered parameter set in the case of a random network. Qualitatively similar results can be observed for a scale-free network as well.

**Figure 5:**
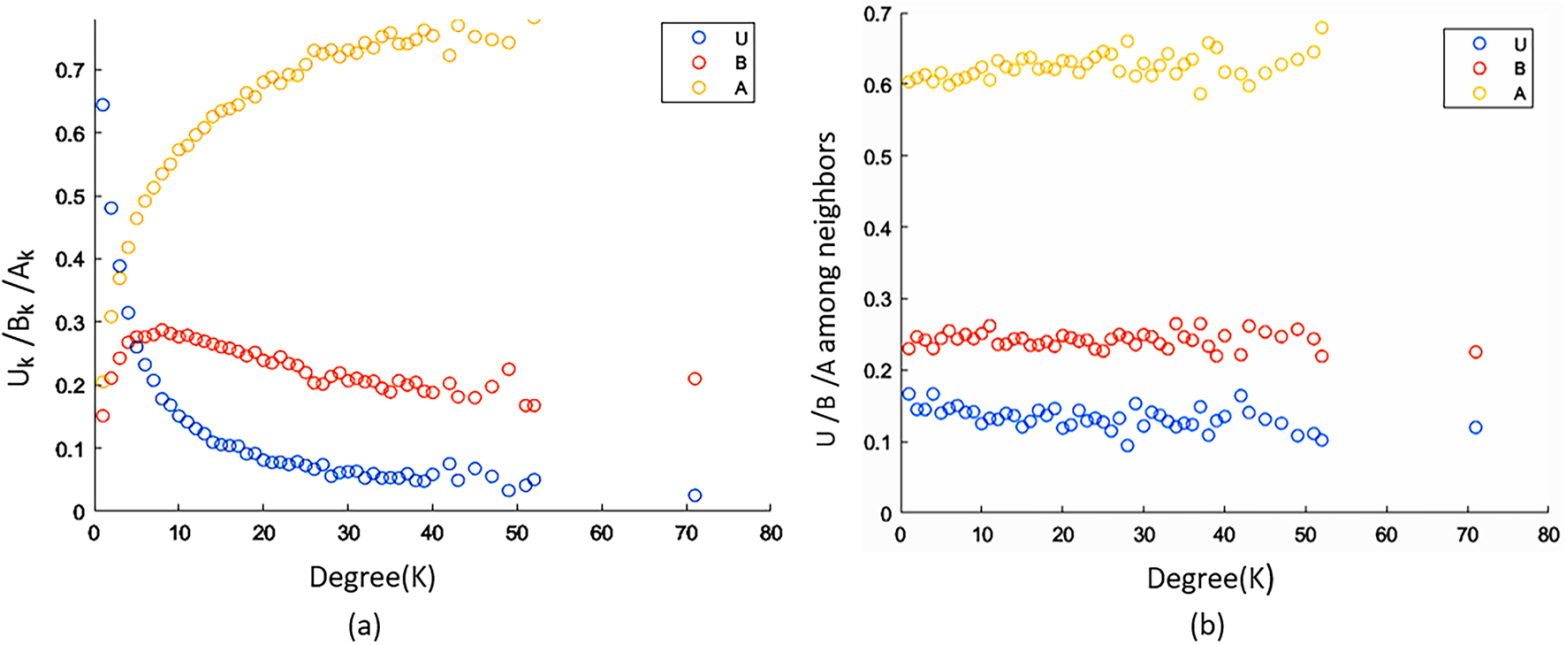
(**a**) Degree-wise fraction of *u*, *b* and *a* with degree *k* in case of a random network at steady-state (**b**) Fraction of *u*, *b* and *a* in the neighborhood of a node with degree *k* in case of a random network. Parameter values are same as Fig, 4.

To understand the impact of the information transmission process from the perspective of nodes with different degrees in the presence of media in a random network, we have plotted the degree-wise steady-state fraction of various classes in Fig. 5a. It can be observed from the figure that the fraction of bootlegger class monotonically increases with degree. Meanwhile, the probability of a node being in an unaware class decreases with the nodal degree. To understand the reason behind this observation, we have plotted the fraction of different classes in the neighborhood of a node in Fig. 5b. It can be seen that this fraction is about the same for all degrees in the random network. This indicates that, statistically, the proportion of neighbors who may disseminate piracy habits to a node is constant across all nodes. But that does not imply that there are the same amount of bootleggers everywhere. It indicates that more bootleggers are surrounding the higher-degree nodes. That is why they are more prone to piracy habit. A similar pattern can also be expected in the case of scale-free networks. Hence, the impact of the contagion process is more significant on higher-degree nodes. These nodes have a high probability of undergoing a transition, making the contagion reach several other nodes connected to them. Hence, they are crucial in the contagion process.

**Figure 6:**
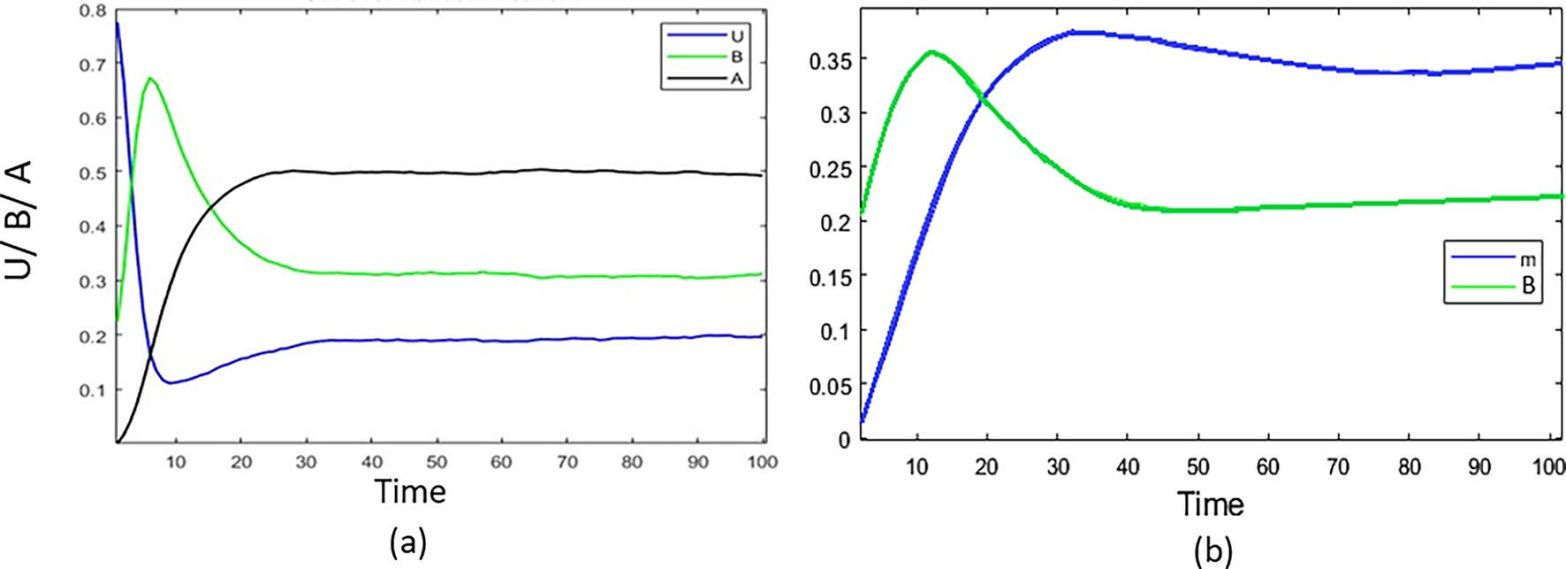
(**a**) Temporal evolution of *u*, *b*, and *a* in the presence of mass media awareness with an initial condition (0.75,0.22,0). (**b**) Similar variation of media level and population of bootleggers with time. Parameter set for both the plots are $\mu =0.05$, $\alpha =2$, $\beta =0.2$, $\rho =0.3$, $\gamma =0.08$, $m_0=4$, $c=5$, $\phi =0.5$, and $\phi _{0}=0.1$.

To show the implication of a media awareness program, we also performed a study on time evolution. We have shown the time evolution of *u*, *b*, and *a* in the case of a random network in Fig 6a for a particular initial condition and seen that after some time, all the population is becoming steady. In Fig. 6b, we have shown the variation of media awareness level and the variation of bootleggers population in a single plot to emphasize that for the success of a media awareness campaign. Interestingly, we observed that the bootlegger population started decreasing once the media effect reach its peak, which implies that media awareness program could be an effective way to control this online piracy contagion.

### Simulation over real networks

Here, we have applied the internet piracy contagion to a real-world network. Being distinct from any model network structure, real networks are able to demonstrate interesting properties. We have used a network of collaboration amongst jazz musicians, referred as Jazz network in the rest of the text. Here, each musician is a node, and their connection denotes that they played in a band together. The information was gathered from the KONECT (The Koblenz Network Collection) database to comprehend its impact on actual social interaction settings. Similar to the homogeneous analysis and random network study, the temporal evolution in the $u-b$ plane for this piracy endemic scenario on the Jazz network has been shown in Fig. 4c. For another real network collected from KONECT database, similar trends has also been observed. Here, we have chosen to study the dynamics in Gnutella peer-to-peer (P2P) file sharing network which is very appropriate for a study on online piracy and real file sharing transmission. All the results related to the steady-state values of different classes under the same parameter set (as per Fig. 4), for homogeneous, random, and for the real network have been listed in Table 2. Here, it is essential to observe that the steady-state values in homogeneous and heterogeneous populations qualitatively resemble each other quite a bit. Even though the exact numbers vary depending on the topology and features in real networks, the physical interpretations and conclusions based on a homogeneous situation are unquestionably accurate nonetheless. Furthermore, in both situations, the impacts of the rate parameters on the contagion process are similar.

**Figure 7:**
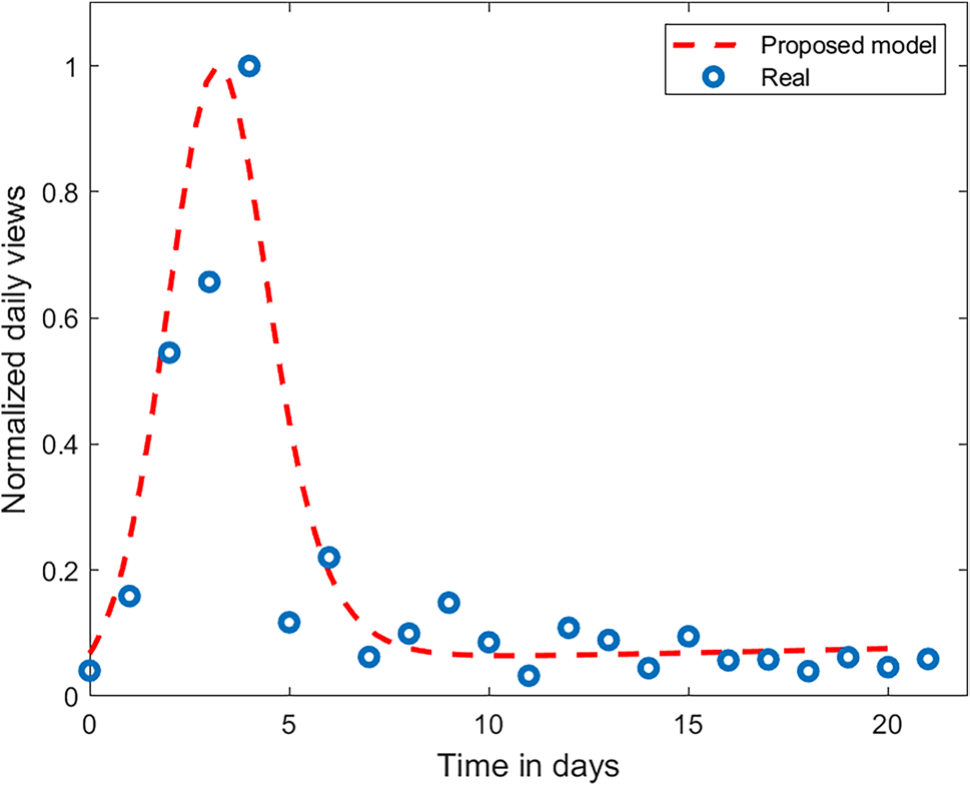
The proposed model fitted to the daily viewing data for a trending YouTube video using parameter values $\mu =0.05$, $\alpha =2$, $\beta =0.001$, $\rho =0.4$, $\gamma =0.4$, $m_0=5$, $c=5$, $\phi =0.7$, and $\phi _{0}=0.1$.

### Parameter validation using real transmission

To study a real transmission scenario, we have analysed the trend of a YouTube video (https://youtu.be/SWmufgTp6EQ?si=UJLA1-TxJQbahleW), titled, “Why Piracy Will Never Stop”. We have chosen this video for its pro-piracy content, which suits our study perfectly. We observed the number of views of the video on a daily basis: from the date of video upload, through the peak, till the cumulative views reached saturation. We fitted the model parameters for the information transmission dynamics and estimated the parameter values. We found the best-fit parameter values for the data and reported it in Fig. 7. We note that the parameters fall into the same range as per our rest of the study, which confirms the validity of our parameter selection.

## Conclusion and discussion

Nowadays, online piracy, i.e., the illegal act of copying, duplicating, and sharing digital work without the permission of copyright holders, is becoming a burning issue globally for the digital industry. Organisations and businesses, frequently in collaboration with the government, have used a variety of anti-piracy tactics, such as innovation, awareness, and law enforcement, to safeguard intellectual property and boost lawful sales. However, there is minimal proof that anti-piracy policies have been effective in bringing down the amount of piracy, despite several attempts to do so. Industry lawsuits have forced the closure of several of the most well-known file-sharing websites. But even after the legal threats, P2P site traffic volume did not dramatically decline. In this ground, we study the effectiveness of a dual-strategy of peer awareness added with positive media campaigns to fight piracy using mathematical tools.

In this paper, we aim to model the social phenomena of online piracy, which exhibits epidemic-like transmission due to its reliance on peer influence. In our study, the online piracy habit has been modeled and analyzed from the perspective of the effectiveness of mass media campaigns in developing awareness among individuals. We used ODE models and graph-theoretical treatment with differential equations on the network to figure out ways to control the habit of piracy in society. The presence of media campaigns helps maintain the number of people aware of the population and directly restricts the spread of this piracy habit. We discovered the parameter threshold regulating piracy states and detected the bifurcations in the system.

To examine the similarities and differences in both the mean-field and network approaches related to this piracy dynamic, we used both homogeneous and heterogeneous methodologies. Though several attempts are made to analyze outbreaks independently using homogeneous and heterogeneous approaches, the similarities and dissimilarities between these approaches must be better explored. Upon first inspection, both these approaches seem different. Homogeneous modeling is based on differential equations and strict assumptions about uniform mixing and contagion, whereas heterogeneous analysis is based on simulations carried over a heterogeneous network structure. Conversely, homogeneous analysis is expected to run fast, whereas heterogeneous analysis may take hours to produce results, depending on the network’s size and the interaction complexity. Numerical simulations for this dynamics were conducted on random and actual online social networks to demonstrate the presence of online piracy prevalence and control. Apart from detailed mathematical and computational analyses, similarities and dissimilarities between the two approaches have also been explicitly quantified. While certain physical information can be achieved from both approaches, each explicitly reveals specific characteristics about the information flow in society, which can be crucial to developing promotional or inhibiting activities. Moreover, we show that media campaigns and positive peer influence can work hand-in-hand to spread awareness and diminish piracy. Importance of high-degree network hubs has also been established.

We should note that there could be certain aspects, inherent to the social network on which the dynamics is going on, that might affect the accuracy of the model. Real networks could be extremely sparse or clustered in cases, and might affect the model dynamics, which can be further explored in a future study. Moreover, as media campaigns always have associated cost, our findings show that if the level of media is adjusted proportionally with population of bootleggers in the society, then an optimal expenditure on campaigning can be achieved, while attaining the target suppression of piracy. This finding may have direct implications on policy making against digital piracy and can be studied using data-driven models.

## Data Availability

The datasets analyzed during the current study are available in the KONECT repository, http://konect.cc/networks/arenas-jazz/.
